# A semi-dwarf and late-flowering Koshihikari d60Hd16: development, productivity, and regional suitability revealed by correlation-based network analysis

**DOI:** 10.3389/fpls.2024.1443149

**Published:** 2025-03-03

**Authors:** Motonori Tomita, Hiroshi Honda

**Affiliations:** ^1^ Research Institute of Green Science and Technology, Shizuoka University, Shizuoka, Japan; ^2^ Honda Biotech. Laboratory, Utsunomiya-Shi, Tochigi, Japan

**Keywords:** rice, semidwarfing gene, *d60*, late-flowering gene, *Hd16*, isogenic genome, traits, production

## Abstract

**Background:**

Breeding rice varieties that are resilient to climate change and optimizing the cultivation methods for each developed variety are challenging issues in global food demands.

**Methods:**

In this study, the late-flowering gene *Hd16* of Koganebare was introduced into Koshihikari through backcrossing to create ‘Koshihikari Hd16’. It was then crossed with ‘Koshihikari d60’ to develop an isogenic line of Koshihikari containing both *Hd16* and *d60*. Productivity tests were conducted in nine prefectures in Japan for two homogeneous rice genotypes: *Hd16* (late flowering) and *d60Hd16* (short culm and late flowering). By analyzing the relationship among genotypes, traits, and accumulation temperatures, we reexamined the characteristics of each genotype and inferred the optimal growing areas.

**Results:**

Correlation-based network analysis between yield, grain quality, and taste value, as well as other traits, showed that quality was negatively correlated with panicle length (*r* = 0.36) and 1,000-grain weight (*r* = 0.43), and yield was strongly positively correlated with 1,000-grain weight (*r* = 0.66). The *d60* genotype was negatively correlated with culm length (*r* = −0.82) and lodging degree (*r* = −0.58). These correlations were supported by partial correlation analysis, and significant differences compared with the wild-type were identified. Principal component analysis revealed that Yamanashi and Ehime, which provided long panicle and culm lengths to ‘Koshihikari d60Hd16’, were suitable in terms of yield; on the other hand, Shimane, which is warmer and produced shorter panicle and culm lengths, was suitable in terms of eating quality. Moreover, Koshihikari d60Hd16, the late-flowering and semi-dwarf strain, could express traits that are less prone to lodging while maintaining the same quality and yield as the wild type.

**Conclusion:**

Thus, the *d60* and *H16* genotypes express stable traits adapted to a wide range of Japanese climatic conditions and growing environments. This study provides fundamental information for the promotion of new smart agriculture, in which improved varieties are deployed in different regions with different climatic conditions.

## Introduction

Rice is cultivated worldwide, particularly in Asia, and is one of the world’s top three grains, with an annual yield of 680 million tons, alongside corn (1.73 billion tons) and wheat (680 million tons) (Chauhan et al., 2017); therefore, stable production is crucial. The Earth has warmed by approximately 1.0°C from pre-industrial levels, and temperatures are predicted to rise another 1.5°C between 2030 and 2052 (JMA, 2024). In the sixth evaluation report of the Intergovernmental Panel on Climate Change (IPCC), global warming is expected to bring about an increase in the frequency of strong tropical cyclones, and there are concerns that the damage from heavy rains will be magnified (IPCC, 2023). Global climate change, population growth, and trade liberalization threaten to disrupt the current agricultural system, which could lead to food problems. Therefore, the molecular genetic breeding of cereals suitable for different climatic conditions is positioned as a challenging issue to be resolved.

Thus far, the “Green Revolution” has contributed to improving the lodging resistance of rice (Khush, 1999; Hergrove and Coman, 2016). The gene contributing to rice “Green Revolution” was identified as *sd1* on the long arm of chromosome 1 (Cho et al., 1994a, b; Maeda et al., 1997), encoding a defective C20-oxidase in the gibberellin (GA) biosynthesis pathway (GA 20-oxidase, OsGA20ox2) (Monna et al., 2002; Sasaki et al., 2002; Spielmeyer et al., 2002), and mutations in the GA 20-oxidase gene lead to disruptions at a late stage of the GA pathway (Sasaki et al., 2002). The *sd1* gene confers no detrimental effects on the grain yield (Hedden, 2003a, b; Tomita and Ishii, 2018). However, the trend of rice production has now begun to plateau (Hergrove and Coman, 2016). Surprisingly, semi-dwarf rice varieties have been developed independently using different native varieties or artificially induced mutant lines as the mother plants have the same *sd1* loci. This narrow gene pool of semi-dwarfness has led to the reduced genetic diversity of rice (Chang and Vergara, 1972; Chang et al., 1985; Hedden, 2003b). To prepare for future increases in population and the risk of crop damage due to climate change, there is a demand for a “New Green Revolution” through renewed genetic improvements instead of *sd1*.

To identify a novel alternative gene to *sd1*, Tomita et al. (1989) conducted gene analyses on Hokuriku 100, a mutant line with 15 cm and a 20% shorter culm than that of Koshihikari. Hokuriku 100 was developed through a large-scale mutation breeding by exposing Koshihikari to 20 kR of gamma radiation with ^60^Co (Samoto and Kanai, 1975). Tomita et al. (1989, 1996) discovered a unique heredity of a novel semi-dwarf gene, *d60*, with the gametic lethal gene *gal*, which complements *d60*, to cause gametic lethality. Moreover, an isogenic line was obtained by integrating both the *d60* and *sd1* derived from Jukkoku (Tomita, 2009; Naito and Tomita, 2013) in the genetic background of Koshihikari (Tomita, 2012). The d60sd1 line became a double-recessive dwarf, indicating that *d60* is functionally independent of *sd1* and is not related to the GA1 biosynthesis pathway (Tomita, 2012). Above all, *d60* is expected to diversify semi-dwarf breeding as a novel alternative to *sd1* (Tomita and Ishimoto, 2019).

Koshihikari is additionally suffering from poor filling and widespread yield reduction caused by high temperature such as heat waves. When the average daily temperature exceeds 23°C–24°C during the 20 days after heading, a white immature grain arises (Hirano and Sano, 2000; Umemoto and Terashima, 2002; Morita, 2005; Yamakawa et al., 2007). Both white-back and milky-white immature grains arise at 27°C, white-back immature grains occur at 30°C, and milky-white immature grains frequently occur at 33°C (Tashiro and Wardlaw, 1991). The recent heat waves caused 170,000 tons of high-temperature damage, namely, deterioration of the rice quality, which was widespread and reached 21% of the total production volume (MAFF, 2019). This is because the leading variety Koshihikari, which comprises 37.3% of the rice acreage in Japan (Rice Stable Supply Organization, 2024), flowers and ripens in the high-temperature phase in August. Rice industries have been strongly requiring late-flowering varieties instead of Koshihikari to avoid high-temperature ripening. Tomita et al. (2022) identified the late-flowering gene *Hd16* from Isehikari, which was identical to that from Nipponbare (Hori et al., 2013). If Koshihikari with both the *Hd16* and *d60* genes could be developed, it would be an effective countermeasure against damage from typhoon-induced collapse and reduced production due to high temperatures.

Therefore, this study attempted to develop two lines, a late-flowering line Hd16 and a short-stalked and late-flowering line d60Hd16, to establish a Koshihikari line that is resilient to climate change. In addition, a productivity test was conducted in nine prefectures in Japan to evaluate performance. Currently, smart agriculture using existing genotypes is being conducted to maximize yield and quality; however, knowledge on the best regions and farming methods for each genotype is limited, and it is important to infer suitable cultivation areas by understanding the correlation between traits, genotypes, and environmental factors. Therefore, in this study, the relationship among genotypes, traits, and integrated temperatures was analyzed using statistical analyses such as correlation analysis, partial correlation analysis, principal component analysis (PCA), and multiple comparison analysis in order to reexamine the characteristics, regional suitability, and productivity of each genotype and to estimate the optimal growing region for each genotype. The study provides fundamental information for the development of new smart agriculture practices to deploy improved genotypes in regions with different climatic conditions.

## Materials and methods

### Development of Koshihikari d60Hd16

**Figure 1:**
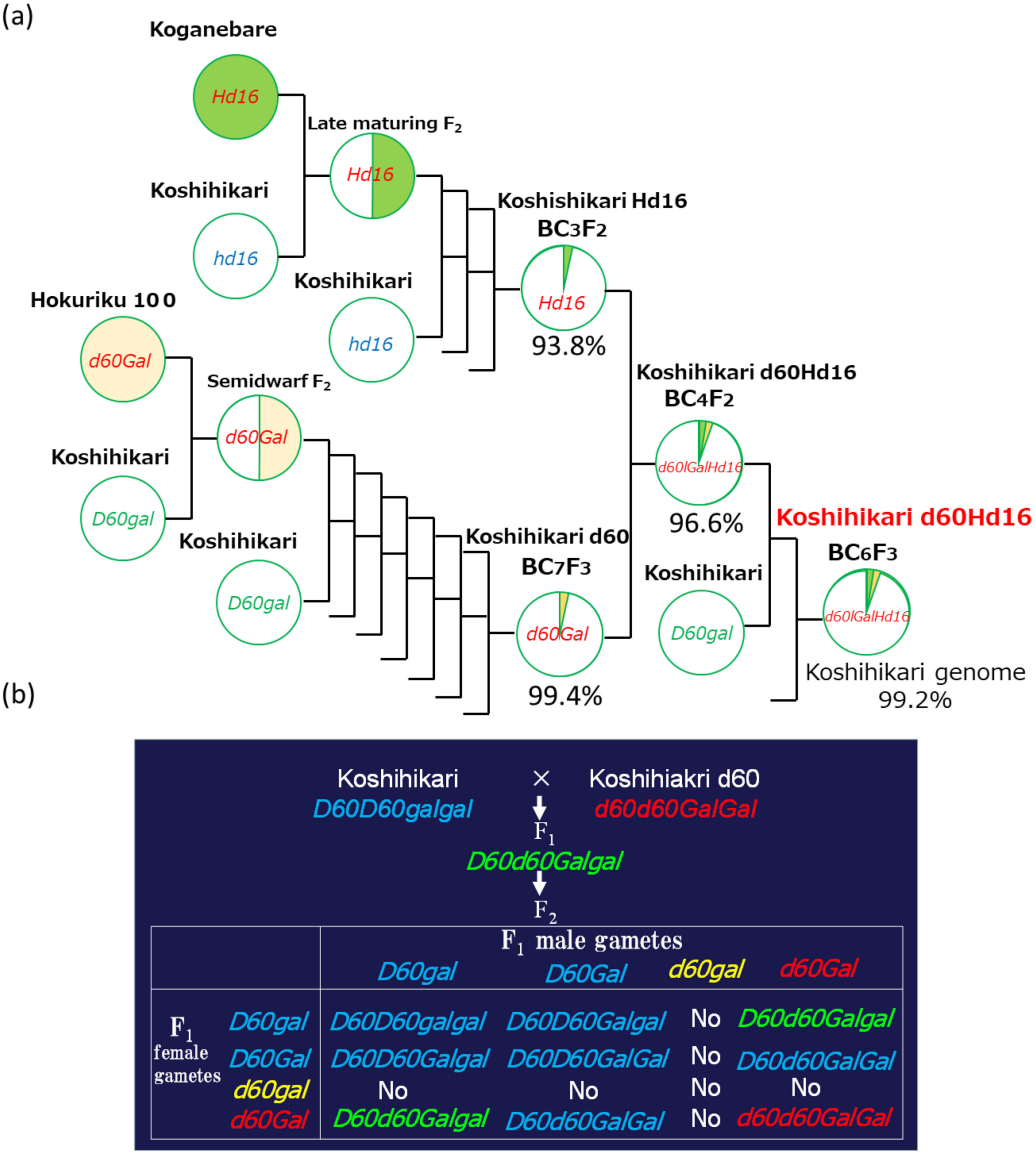
Phylogenetic process of [Koshihikari*2///Koshihikari d60//Koshihikari*3/[Koshihikari x Koganebare F_2_ late-flowering type]] BC_6_F_2_. **(A)** Three backcrosses were conducted with Koshihikari as a recurrent parent using a late-flowering plant compared with Koganebare, which was segregated in F_2_ of Koshihikari x Koganebare as a non-recurrent parent. The fourth backcrossing with Koshihikari d60 was conducted using a late-flowering plant that was segregated from Koshihikari*3/[Koshihikari x Koganebare F_2_ late-flowering type] BC_3_F_2_. The fifth backcrossing with Koshihikari was conducted with a late-flowering segregant in B_4_F_2_, which was considered *d60* homozygous. A semi-dwarf and late-flowering segregant in BC_5_F_2_ selected as *d60* homozygous was backcrossed with Koshihikari six times, and semi-dwarf and late-flowering BC_6_F_2_ segregants were selected as *d60* homozygous and fixed as *d60Hd16* homozygous in BC_6_F_3_. **(B)** From BC_4_F_2_ to BC_6_F_2_, the *d60* allele was segregated at a ratio of 4*D60D60*:4*D60d60*:1*d60d60*, according to complementally gametic lethal with *gal* (Tomita, 1996; Tomita, 2012; Tomita and Tanisaka, 2019).

Three backcrosses were conducted with Koshihikari (Etsunan no. 17) as the recurrent parent using a late-flowering plant compared with Koganebare (Aichi no. 40) that was segregated in F_2_ of Koshihikari × Koganebare as the non-recurrent parent (**Figure 1**). As Koganebare is a late-flowering variety descended from Nipponbare, we hypothesized that the related late-flowering gene would be *Hd16*. The fourth backcrossing with Koshihikari was conducted using the late-flowering plant that was segregated from Koshihikari*3/[Koshihikari × Koganebare F_2_ late-flowering type] BC_3_F_2_. A total of 90 plants of Koshihikari*4/[Koshihikari × Koganebare F_2_ late-flowering type] BC_4_F_2_ were examined. At the same time, 60 plants of Koshihikari d60//Koshihikari*3/[Koshihikari × Koganebare F_2_] BC_4_F_2_ that were back-crossed with Koshihikari d60 were examined (**Figure 1**). The Koshihikari d60 line is an isogenic Koshihikari having *d60* and *Gal*, which was developed through seven times of continuous backcrossing with a recurrent parent, Koshihikari, using a non-recurrent parent of the *d60* homozygous segregant in the F_2_ of Koshihikari × Hokuriku100 (Tomita, 2012). A fifth backcrossing with Koshihikari was conducted with a late-flowering segregant in B_4_F_2_, which was considered *d60* homozygous. The heading date was 64 days, and the culm length was 33.4 cm. A total of 116 plants of [Koshihikari///*d60* Koshihikari//Koshihikari*3/[Koshihikari × Koganebare F_2_ late-flowering type]] BC_5_F_2_ were examined. A semi-dwarf segregant in BC_5_F_2_ was selected as a *d60* homozygous late-flowering plant, followed by a sixth backcrossing with Koshihikari d60. [Koshihikari d60////Koshihikari///Koshihikari d60//Koshihikari*3/[Koshihikari × Koganebare F_2_ late-flowering type]] BC_6_F_2_ was then examined. The heading date and the culm length were investigated in all plants, and late-flowering and semi-dwarf plants that were considered *d60* homozygous were selected. The above developmental process was conducted by M. Tomita from 2008 to 2020 at Tottori University and Shizuoka University. A genetic diagnosis was conducted using the SSR marker RM16089 (chr3: 33.7 Mb), which is closely linked to the late-flowering gene *Hd16*, as well as those described in Tomita et al. (2022).

### Whole-genome sequencing analysis

**Figure 2:**
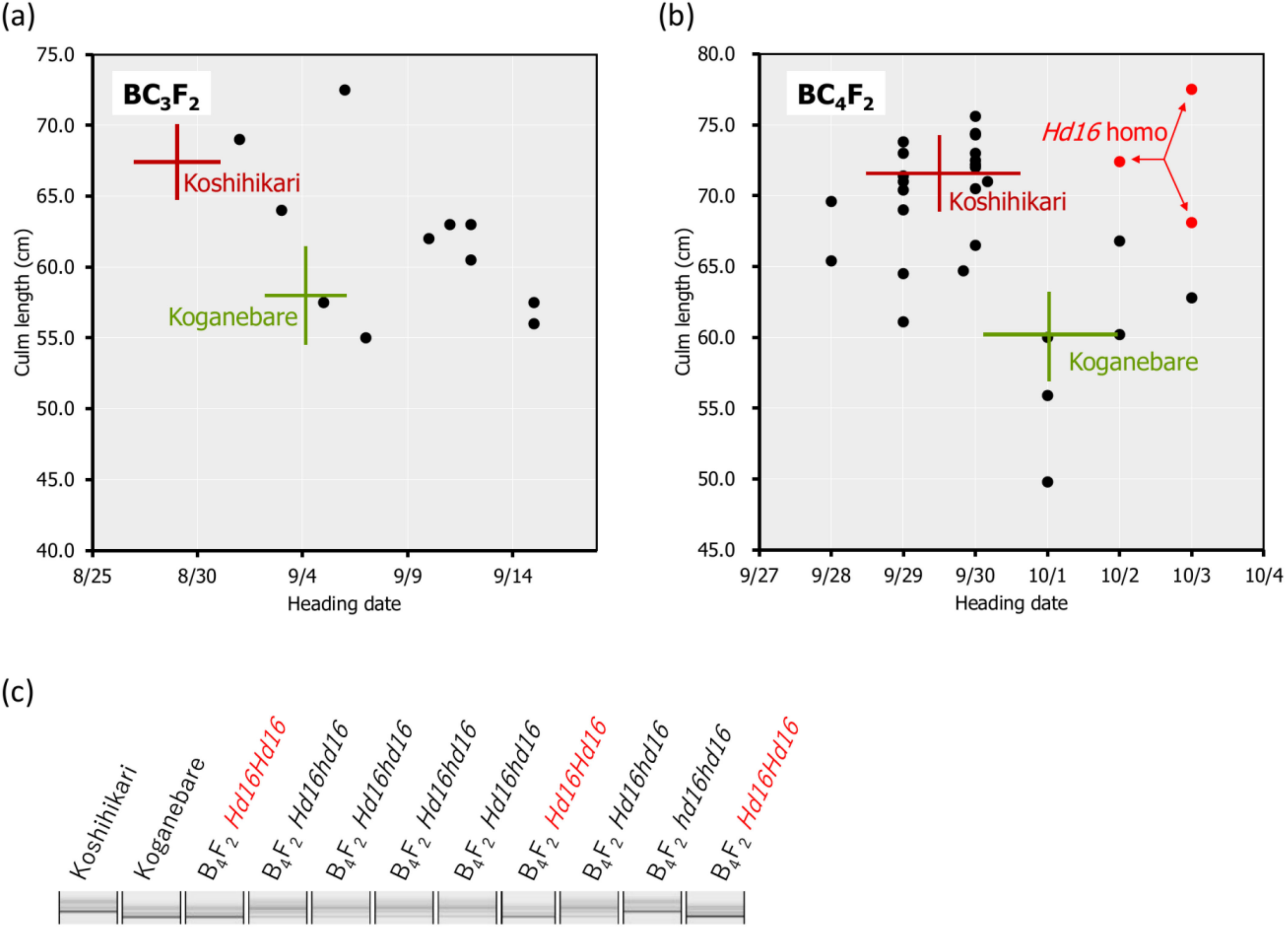
Relationship between heading date and culm length and the diagnosis of *Hd16* using RM16089 in BC_3_F_2_, BC_4_F_2_. In Koshihikari*3/[Koshihikari x Koganebare F_2_ late-flowering type] BC_3_F_2_, which was backcrossed three times with Koshihikari as the recurrent parent using a late-flowering segregant compared with Koganebare in F_2_ of Koshihikari x Koganebare as the non-recurrent parent, the heading dates were distributed from early maturation compared with Koshihikari to late-flowering later than Koganebare **(A)**. The fourth back-crossing with Koshihikari was conducted using the most late-flowering segregant in BC_3_F_2_. Genetic diagnosis was performed using the SSR marker RM16089, which is closely linked to the late-flowering gene *Hd16* in BC_4_F_2_ of Koshihikari*4/[Koshihikari x Koganebare F_2_ *Hd16* semi-dwarf type] **(B, C)**. As a result, BC_4_F_2_ segregated into a ratio of 1 RM16089 homozygous:2 heterozygous:1 RM16089 null type and the RM16089 homozygous; that is, the *Hd16* homozygous line was obtained **(B, C)**. The target genotype is indicated in *red*. The range of culm length and the heading date of each variety are shown as standard deviation using *error bars*.

Whole-genome sequencing (WGS) was conducted for both Koshihikari Hd16 (BC_7_F_4_) and Koshihikari d60Hd16 (BC_8_F_2_), which was integrated with the late-flowering gene *Hd16* and the semi-dwarfing gene *sd1* through eight times of backcrossing into the genetic background of Koshihikari. The leaves were powdered using a mortar and pestle while frozen in liquid nitrogen. The genomic DNA was then extracted from each genetic line with the CTAB method. Genomic DNA was fragmented and simultaneously tagged so that the peak size of the fragments was approximately 500 bp using the Nextera^®^ transposome (Illumina Inc., San Diego, CA, USA). After purification of the transposome with DNA Clean & Concentrator™-5 (Zymo Research, Irvine, CA, USA), the adaptor sequences, including the sequencing primers, for fixation on the flow cell were synthesized at both ends of each fragment using polymerase chain reaction. Subsequently, the DNA fragments were subjected to size selection using AMPure XP magnetic beads (Beckman Coulter, Brea, CA, USA). Finally, qualitative checks using Fragment Analyzer™ (Advanced Analytical Technologies, Heidelberg, Germany) and quantitative measurements with Qubit^®^ 2.0 Fluorometer (Life Technologies, Thermo Fisher Scientific, Inc., Waltham, MA, USA) were performed to prepare a DNA library for next-generation sequencing (NGS). Sequencing was conducted in paired-end 2 × 100 bp on a HiSeq X next-gen sequencer according to the manufacturer’s protocol (Illumina Inc., San Diego, CA, USA). The obtained Illumina reads were firstly trimmed using Trimmomatic (version 0.39) (Bolger et al., 2014) (**Figure 2**). The sequencing adapters and the sequences with low-quality scores on 3′ ends [Phred score (*Q*), <20] were trimmed. Raw Illumina WGS reads were quality checked by performing quality control with FastQC (version 0.11.9; Babraham Institute). Mapping of the reads from Koshihikari Hd16 and Koshihikari d60Hd16 to the Koshihikari genome as a reference was conducted with Burrows–Wheeler Aligner (BWA) software (version bwa-0.7.17.tar.bz2) (Li and Durbin, 2009). Duplicate reads were removed using Picard (version 2.25.5) (http://broadinstitute.github.io/picard), while secondary aligned reads were removed using SAMtools (version 1.10.2) (Li et al., 2009). To identify genetic variations among strains, single nucleotide variant (SNV) detection (variant calling) and SNV matrix generation were performed using GATK version 4.1.7.0 (McKenna et al., 2010).

### Productivity (performance) tests

Productivity tests were conducted at experimental stations in Miyagi, Yamanashi, Shizuoka, Mie, Osaka, Shimane, Ehime, Kochi, and Saga prefectures for Koshihikari, Koshihikari Hd16, and Koshihikari d60Hd16 to determine the following parameters: culm length (in centimeters), panicle length (in centimeters), number of panicles (per square meter), lodging degree, 1,000-grain weight (in grams), protein content (in percent), accumulated temperature (in degree Celsius), grain yield (in kilograms per annum), grain quality, and taste value. Accumulated temperature was treated as an environmental factor and *d60* and *Hd16* as loci. In the tabulation of the results of the trials, the sample name was the combination of the growing region and the variety name (e.g., Yamanashi_d60Hd16). Cultivation of genetic materials was carried out in 2021 and 2022. Seedlings were individually transplanted into a paddy field in mid-July, with a transplanting density of 22.2 seedlings/m^2^ (one seedling per 30 × 15 cm). The paddy field was fertilized with 4.0 kg of basal fertilizer containing nitrogen, phosphorus, and potassium (weight ratio: nitrogen/phosphorus/potassium = 2.6:3.2:2.6), with 4.3 g/m^2^ nitrogen, 5.3 g/m^2^ phosphorus, and 4.3 g/m^2^ potassium across the field. The heading date was recorded as the date the first panicle had emerged from the flag leaf sheath for each plant. Culm length (in centimeters) was measured as the length between the ground surface and the panicle base. For the yield test, after ripening, 10 plants typical of each genotype were sampled twice. The sampled plants were air-dried and were assessed for the following traits: panicle length, number of panicles, number of florets/panicles, proportion of fertile florets, total panicle number, and weight of unmilled rice/1,000 grain. The yield of unpolished rice was calculated using the following equation: Yield of unmilled rice (g/m^2^) = (number of panicles/m^2^) × (number of florets/panicle) × (proportion of fertile florets) × (weight of unmilled rice/grain). Grain quality was classified into nine grades: from 1—excellent or good to 9—especially bad or low quality. The eating taste was evaluated into seven grades of organoleptic assessment by panelists, while the protein contents were determined using Infratec 1241 (VOSS Japan Ltd., Tokyo, Japan).

### Optimal growing regions for each genetic line

The optimal growing regions for each genetic line were defined as areas with relatively good grain yield, grain quality, and taste values in the assessed areas.

### Correlation network analysis

Correlation network analysis was conducted to visualize the correlations between the items of the productivity tests and to understand their relationship in the big picture. Pearson’s correlation coefficients and partial correlation coefficients were used as indicators of correlations in the integrated analysis of all genotypes. The partial correlation coefficient is an indicator of how two variables are correlated “without the influence of the specified variable.” After first obtaining the correlation and partial correlation coefficients, the strength of the correlation coefficient between each variable was plotted as a graph of the network structure using qgraph, an R package. The relationships between variables with correlation coefficients of 0.3 or higher between variables were illustrated.

Although correlation network analysis is suitable for a broad understanding of the correlation between each variable, it is not sufficient to show the values of the correlation coefficients between variables, the statistically significant differences, and the certainty of the analysis. Therefore, a pairwise scatter plot was constructed to visualize the strength of the proportional relationship between each variable when each result of the productivity test was used as a variable. Pearson’s correlation coefficients were used to analyze the correlations, and tests of correlation coefficient and statistical significance were carried out. The analysis was carried out using the R package PerformanceAnalytics, which can simultaneously draw histograms for each variable, Pearson’s correlation coefficients, and statistically significant differences between each variable and scatter plots between variables.

### Principal component analysis

χCorrelation network analysis and PCA analyze the correlations between variables, but do not clarify the relationship between each growing region and variable. Therefore, a PCA was conducted to identify the relationship between each growing region (each province) and the variables that characterize it. The traits and environmental factors contributing to the separation of each sample when grouped by production area and genetic line were presented as biplots to provide insights into the optimal production area. The R package factoextra was used for the analysis.

### Multiple comparison analysis

The distributions of each trait, environmental factor, and target trait were visualized as a violin plot. The statistical significance of the differences in the means was analyzed using a pairwise *t*-test. The R package rstatix was used for the analysis.

## Results

### Development of the late-maturing Koshihikari-type isogenic line “Koshihikari Hd16”

In BC_3_F_2_, which was backcrossed three times with Koshihikari as the recurrent parent using a late-flowering segregant compared with Koganebare in F_2_ of Koshihikari × Koganebare as the non-recurrent parent (**Figure 1**), the heading dates were distributed from early-flowering compared with Koshihikari to late-flowering later than Koganebare (**Figure 2A**). This segregation was similar to the 1 Koshihikari-type early-flowering plant:2 medium-flowering plants:1 two weeks later-flowering plants than the Koshihikari caused by the segregation of the *Hd16* allele from B_3_F_2_ to BC_6_F_2_ (Tomita et al., 2022), in which Koshihikari was continuously backcrossed using a late-flowering segregant in F_2_ of Koshihikari × Isehikari, a non-recurrent parent. Koganebare, which derived from the cross of Nipponbare × Kiho, is a late-flowering variety with the same ripening as Nipponbare. Therefore, Koganebare is presumed to harbor the *Hd16* derived from Nipponbare (Hori et al., 2013). The fourth backcrossing with Koshihikari was conducted using the most late-flowering segregant in BC_3_F_2_. The genetic diagnosis was conducted using the SSR marker RM16089, which is closely linked to the late-flowering gene *Hd16* in BC_4_F_2_ of Koshihikari*4/[Koshihikari × Koganebare F_2_ *Hd16* late-flowering type] (**Figures 2B, C**). As a result, BC_4_F_2_ segregated into a ratio of 1 RM16089 homozygous:2 heterozygous:1 RM16089 null type and the RM16089 homozygous; that is, the *Hd16* homozygous line was obtained (**Figures 2B, C**).

### Development of the late-flowering and semi-dwarf Koshihikari-type line “Koshihikari d60Hd16”

**Figure 3:**
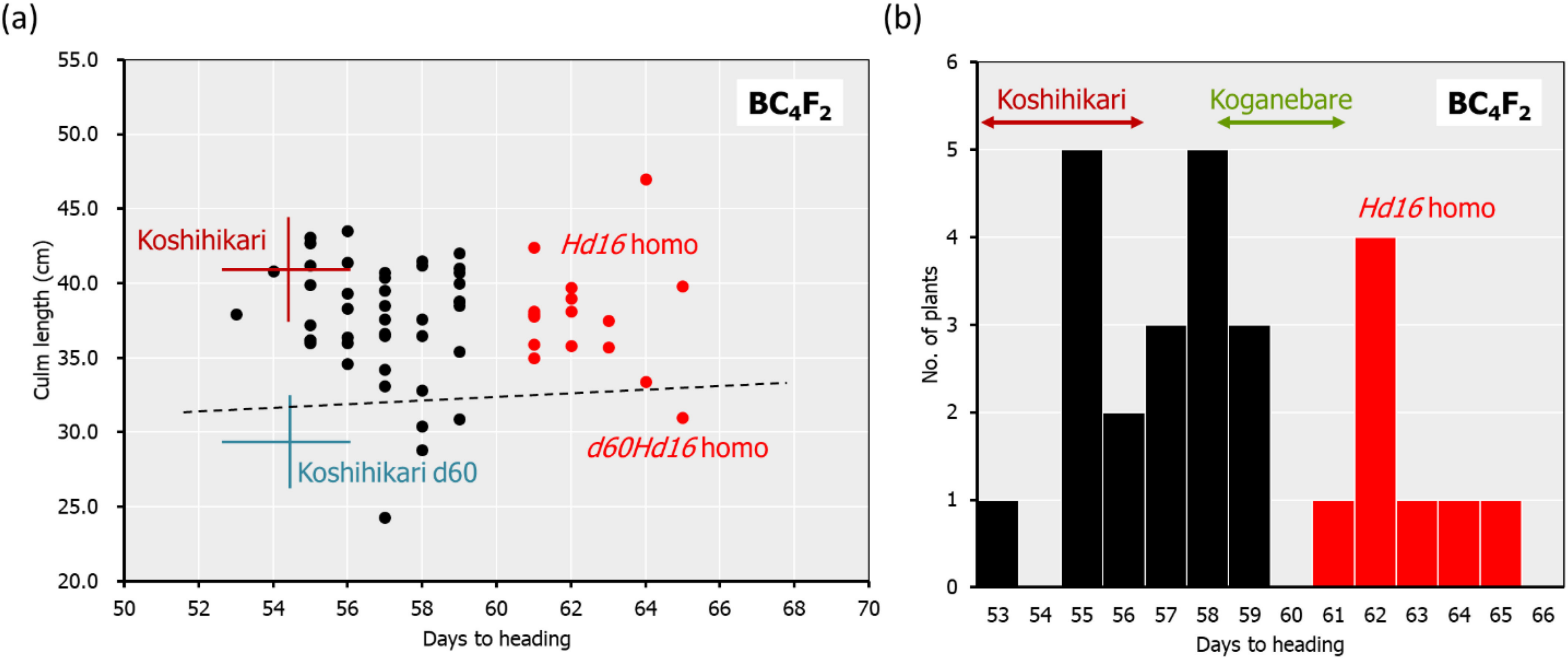
Relationship between heading date and culm length in BC4F2 of Koshihikari d60//Koshihikari*3/[Koshihikari x Koganebare F2]. In BC_4_F_2_, long-culm plants (33–46 cm) and semi-dwarf plants similar to Koshihikari *d60* (25–32 cm), which were considered to be *d60* homozygous plants, were segregated at a ratio of 35:5 **(A)**. The segregants *under the hash line* indicate the *d60* homozygous plants. This ratio fitted the theoretical ratio of 8:1 caused by the segregation of the *d60* and *gal* alleles. The heading date was distributed with a triadic peak at a ratio of 7 *hd16* homozygous early-flowering type:12 *Hd16hd16* heterozygous medium-flowering type:8 *Hd16* homozygous late-flowering type (*red*) = 1:2:1 (χ^2^ = 0.407, 0.80 < *p* < 0.85), and this ratio was compatible with the theoretical ratio of single-gene inheritance **(A, B)**. The *Hd16* homozygous plants are indicated in *red*. The range of culm length and the days to heading for each line are shown as standard deviation using *error bars*.

Backcrossing with Koshihikari as the recurrent parent was performed three times using the Koganebare-type late-flowering maturing segregant in F_2_ of Koshihikari × Koganebare as the non-recurrent parent (**Figure 1**). Koshihikari d60 was crossed with the latest flowering segregant in Koshihikari*3/[Koshihikari × Koganebare F_2_] BC_3_F_2_. In BC_4_F_2_, long-culm plants with culm lengths similar to Koshihikari (39–46 cm), intermediate plants (33–38 cm), and semi-dwarf plants (25–32 cm) similar to those of Koshihikari d60 plants were segregated. The former two long phenotypes and the last semi-dwarf phenotype, which was considered *d60* homozygous, were segregated at a ratio of 35:5 (**Figure 3A**). This ratio fitted the theoretical ratio of 8:1 caused by the segregation of the *d60* and *gal* alleles. The heading date was distributed with a triadic peak at a ratio of 7 *Hd16* homozygous early-flowering type:12 *Hd16hd16* heterozygous medium-flowering type:8 *Hd16* homozygous late-flowering type = 1:2:1 (*χ* ^2^ = 0.407, 0.80 < *p* < 0.85). This ratio was compatible with the theoretical ratio of single-gene inheritance (**Figure 3B**).

**Figure 4:**
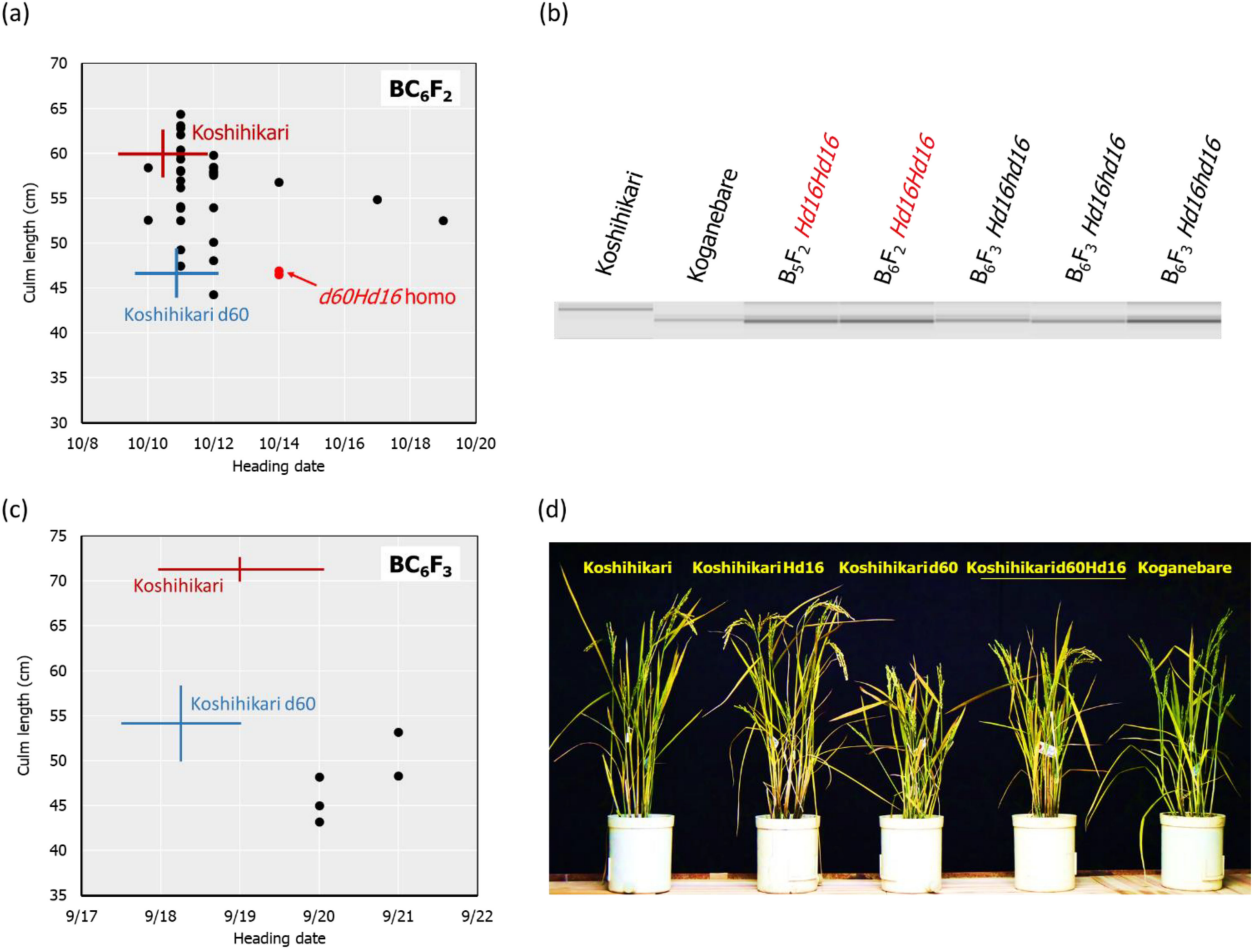
Relationship between heading date and culm length, diagnosis, and morphological alterations in BC_6_F_2_ and BC_6_F_3_. In BC_6_F_2_, long-culm plants (56–65 cm) compared with Koshihikari or intermediate plants and semi-dwarf plants (44–49 cm) compared with Koshihikari *d60* were segregated at a ratio of 26:5, and this ratio was fitted to the theoretical ratio of 8:1 **(A)**. Genetic diagnosis for *Hd16* was conducted using the SSR marker RM16089, and a *d60Hd16* homozygous semi-dwarf BC_6_F_2_ plant (46 cm, October 14) was selected **(A, B)**. The target genotype is indicated in *red*. The progeny line BC6F3 was fixed in *d60Hd16* homozygous **(B, C)**. The semi-dwarf and late-flowering Koshihikari line having *d60* and *Hd16* homozygous acquired in BC_6_F_3_ was 14.6 cm (16%) shorter than Koshihikari and was characterized with a deep green color **(D)**. The culm length range and the heading date of each variety are shown as standard deviation using *error bars*.

The *d60* homozygous late-flowering segregant in BC_4_F_2_ with days to heading of 64 days and a culm length of 33.4 cm was backcrossed five times with Koshihikari. In BC_5_F_2_, long-culm plants (58–71 cm) similar to Koshihikari and semi-dwarf plants (~54 cm) similar to Koshihikari d60 were segregated at a ratio of 21:1. This ratio was fitted to the theoretical ratio of 8:1 caused by the *d60* and *gal* alleles. The genetic diagnosis of *Hd16* was conducted using the SSR marker RM16089, and a *d60Hd16* homozygous semi-dwarf plant (54 cm, October 7) was selected. Furthermore, it was backcrossed six times with Koshihikari. In BC_6_F_2_, long-culm plants (56–65 cm) compared with Koshihikari or intermediate plants and semi-dwarf plants (44–49 cm) compared with the Koshihikari d60 were segregated at a ratio of 26:5. This ratio was in accordance with the theoretical ratio of 8:1 (**Figure 4A**). The genetic diagnosis of *Hd16* was conducted for BC_6_F_2_ and BC_6_F_3_ using the SSR marker RM16089, and a *d60Hd16* homozygous semi-dwarf BC_6_F_2_ plant (46 cm, October 14) was selected (**Figures 4A, B**). In addition, all progenies were fixed with the *d60* homozygous semi-dwarf type in BC_6_F_3_ (**Figures 4B, C**). As a result, Koshihikari d60Hd16 was obtained. The semi-dwarf and late-flowering isogenic line of Koshihikari having the *d60* and *Hd16* homozygous acquired in BC_6_F_3_ was 14.6 cm (16%) shorter than Koshihikari and was characterized with a deep green color (**Figure 4D**).

**Figure 5:**
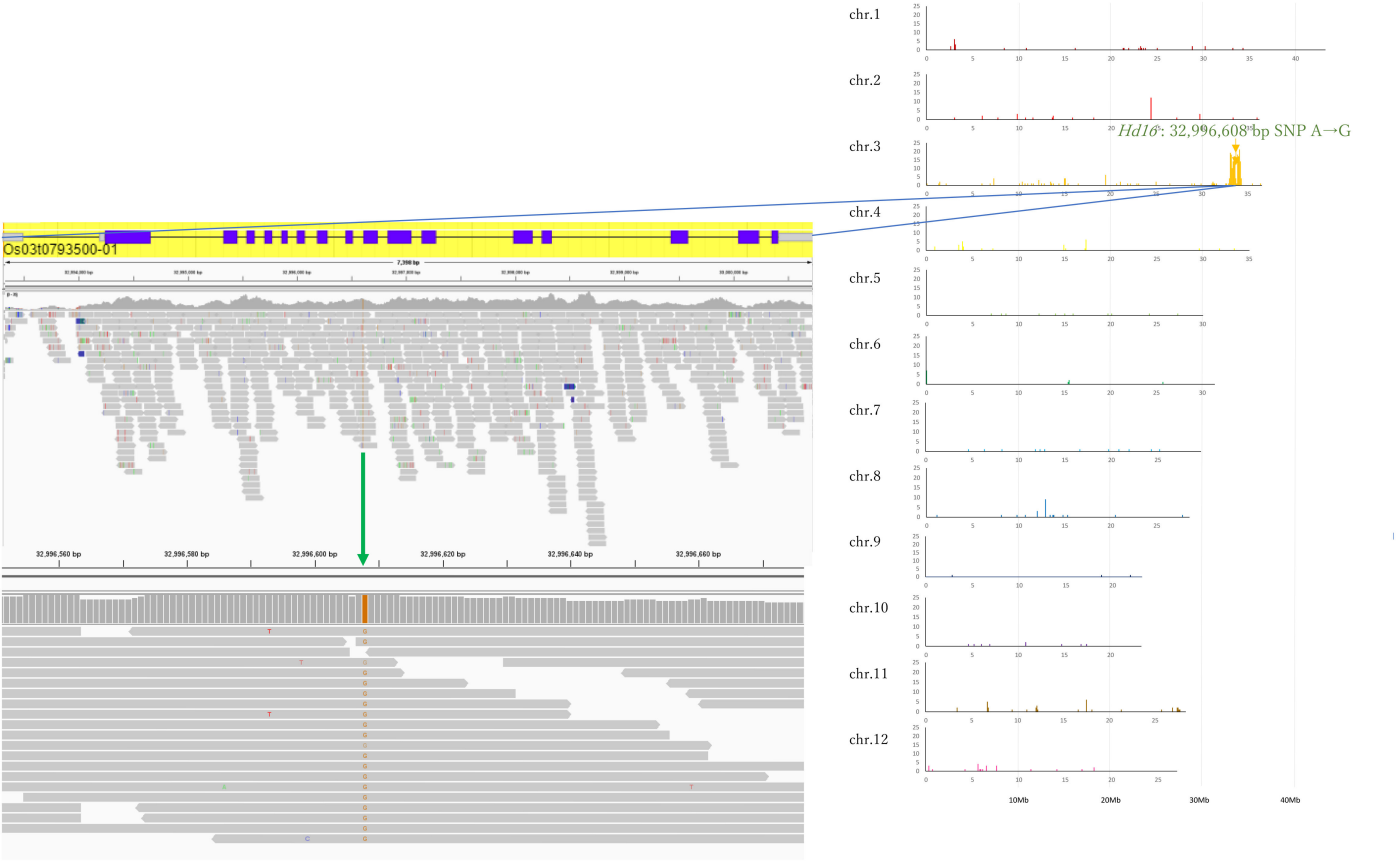
Causative SNP for *Hd16* in Koshihikari d60Hd16. A single SNP from adenine to guanine was detected at 32,996,608 bp in the *Hd16* gene on chromosome 3, which was known as a causative mutation of *Hd16* in Nipponbare.

In Koshihikari d60Hd16 (BC_6_F_3_), SNPs from adenine to guanine were detected in the *Hd16* gene at 32,996,608 bp on chromosome 3, which was known as a causative mutation of *Hd16* in Nipponbare (Hori et al., 2013) and Isehikari (Tomita et al., 2022) (**Figure 5**). Except for the region around *Hd16*, the number of SNPs was less than 10 per 0.1 Mb (**Figure 5**). The results indicated that a large proportion of the rice 12 chromosomes were substituted to the genome of Koshihikari after continuous backcrossing targeting *Hd16*.

### Correlation and partial correlation analysis

**Figure 6:**
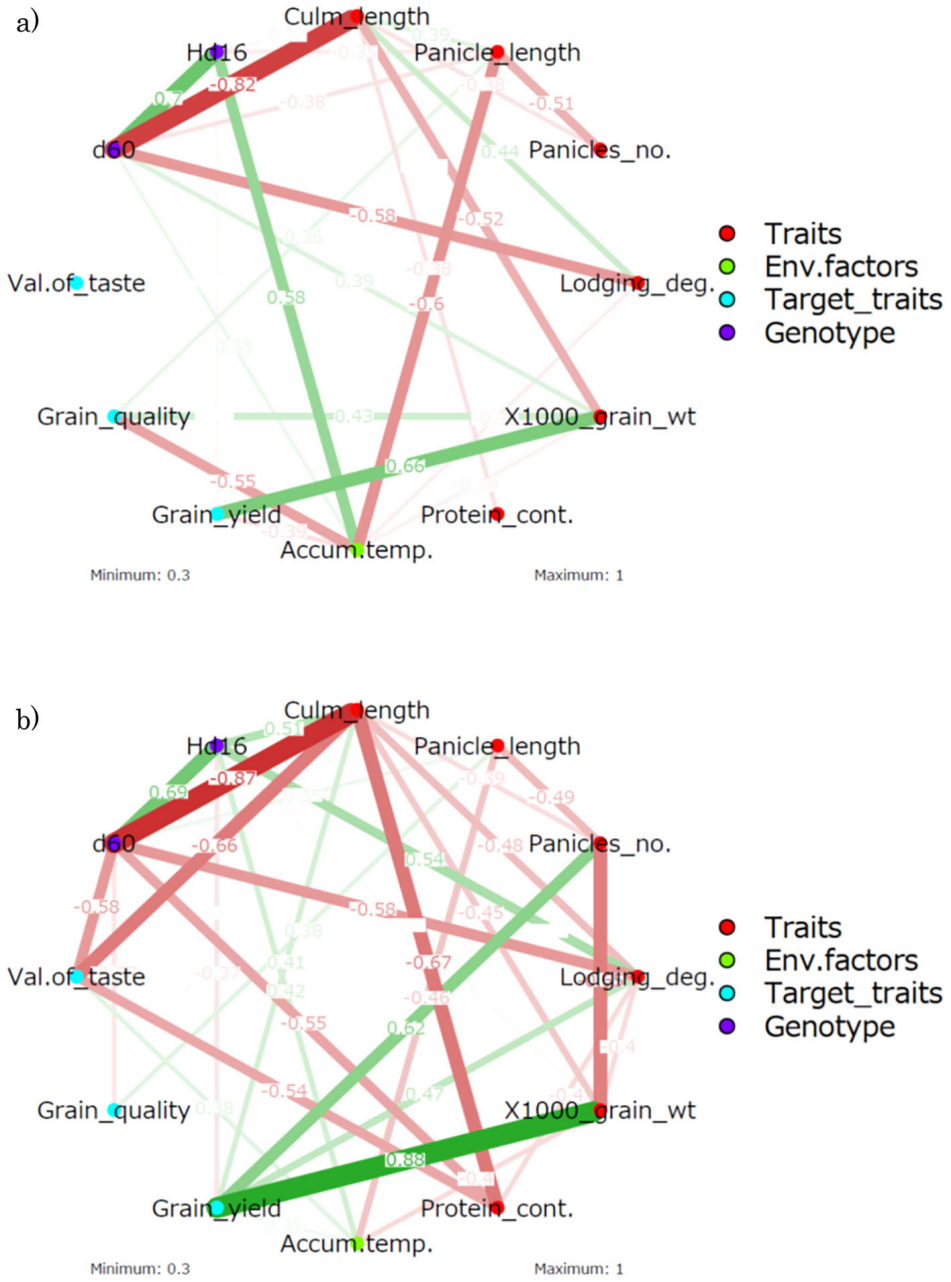
Correlation-based network analysis of the traits, environmental factors, target traits, and genotypes. **(A)** Correlation analysis. **(B)** Partial correlation analysis. *WT*, wild type; *Panicles_no.*, number of panicles; *Lodging_deg.*, degree of lodging; *1,000_grain_wt*, 1,000-grain weight; *Protein_cont.*, protein content; *Accum.temp.*, accumulated temperature. Grain_quality: Grain quality score (small value is high quality). *Values on the edges* indicate Pearson’s correlation coefficients. The *density of the network* indicates the strength of the correlation coefficient, with *red* denoting a negative correlation and *green* denoting a positive correlation.

The results of the productivity trials integrating the three genetic lines are shown in **Supplementary Table S1**. The data for each line were integrated and the relationship among yield, grain quality, taste value, and traits analyzed (**Supplementary Table S1**; **Figure 6**). A positive correlation was found between grain quality score and panicle length in the analysis of correlation coefficients (*r* = 0.36) (Grain quality was negatively correlated with panicle length). Grain yield was strongly positively correlated with 1,000-grain weight in the correlation analysis (*r* = 0.66). These correlations were confirmed in the partial correlation analysis. The positive correlation between lodging degree and culm length (*r* = −0.44) made it easy to understand that the heavier the grain yield, the easier the lodging degree. The correlation analysis did not find a significant correlation for taste value, but the partial correlation analysis showed negative correlations with culm length and protein content (*r*_p_ = −0.66 and −0.54, respectively). A negative partial correlation between culm length and protein content was also observed (*r*_p_ = −0.67).

Subsequently, the relationship between genotype and trait was examined. In the correlation analysis, *Hd16* was negatively correlated with panicle length (*r* = −0.35). However, this negative correlation would be a spurious one as a negative correlation between accumulated temperature and panicle length (*r* = −0.6) was observed and no trend was observed in the partial correlation analysis. Although *Hd16* was positively correlated with accumulated temperature (*r* = 0.58), the Hd16 line was grown in different seasons, resulting in lower accumulated temperatures. In the partial correlation analysis, the correlations between traits should be considered, but positive correlations with culm length (*r*_p_ = 0.51) and lodging degree (*r*_p_ = 0.54) and a negative correlation with grain yield (*r*_p_ = −0.37) were found. These partial correlations may imply potential functionalities of *Hd16* that are not superficially identified.

On the other hand, *d60* was negatively correlated with culm length (*r* = −0.82) and lodging degree (*r* = −0.58). Furthermore, in the partial correlation analysis, *d60* was negatively correlated with culm length (*r*_p_ = −0.87) and lodging degree (*r*_p_ = −0.58) and newly negatively correlated with taste value (*r*_p_ = −0.58) and protein content (*r*_p_ = −0.55). This negative partial correlation with taste value and protein content was interpreted as originating from the negative partial correlation between culm length and taste value and protein content.

The scatter plots between variables and the statistical significance of the correlation coefficients are shown in **Supplementary Figure S2**. It was confirmed that the correlation coefficients were statistically significant at the combinations with high correlation coefficients and that the distribution of the samples was uniform in the scatter plots, thus confirming that there were no issues with the reliability of the correlation analyses.

### Principal component analysis

**Figure 7:**
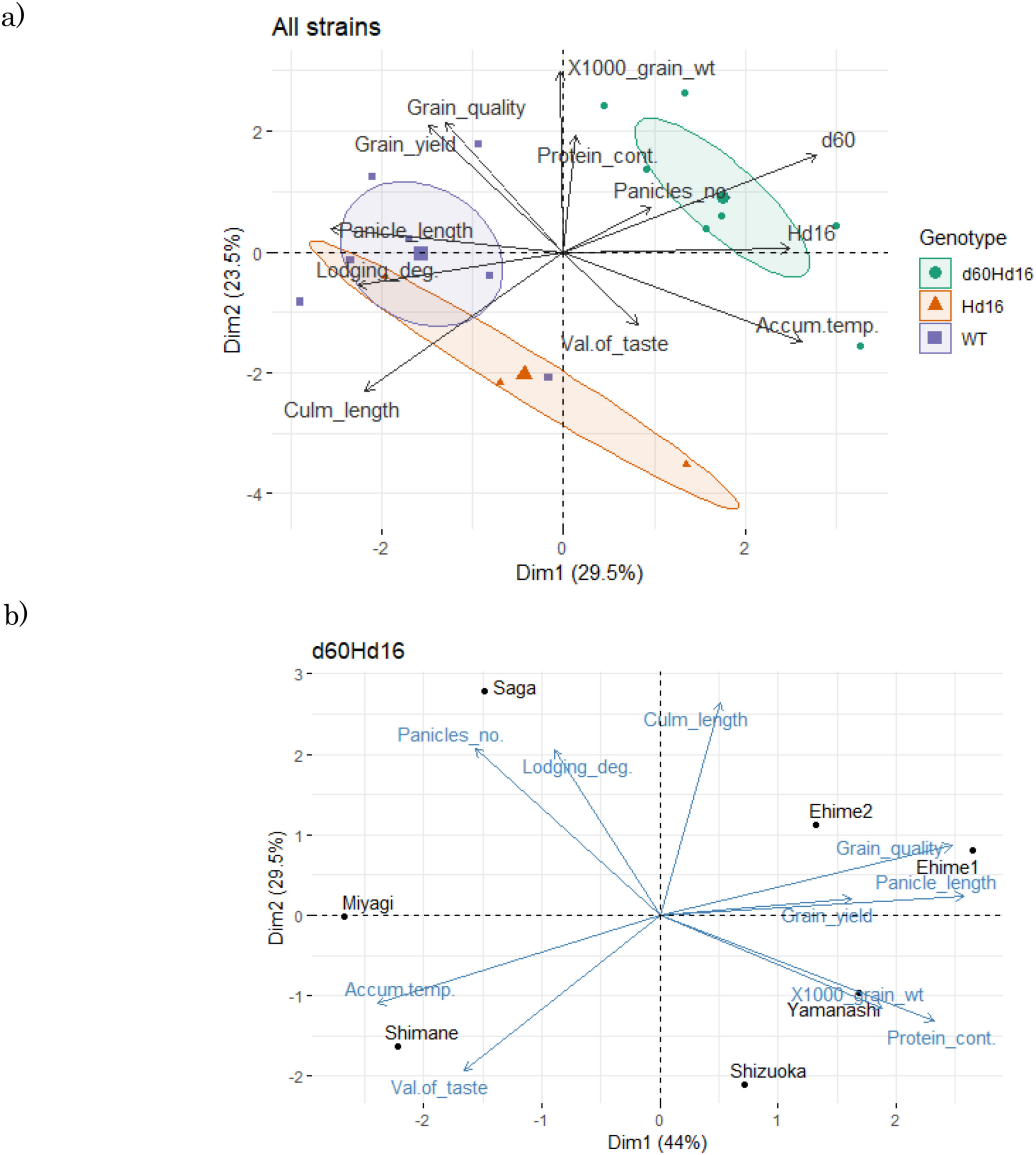
Principal component analysis of the feature differences among genotypes. **(A)** Data on the three strains. **(B)** Data on *d60Hd16*. *Dim1*, dimension 1 (principal component 1); *Dim2*, dimension 2 (principal component 2); *WT*, wild type; *Panicles_no.*, number of panicles; *Lodging_deg.*, degree of lodging;, *1000_grain_wt*, 1,000-grain weight; *Protein_cont*., protein content; *Accum.temp.*, accumulated temperature. Grain_quality: Grain quality score (Only this arrow shows a negative correlation).

The results of the PCA of the productivity tests integrating the three genetic lines showed that they were arranged separately according to genotype based on principal components 1 and 2 (**Figure 7A**). The wild-type genotypes tended to give longer culm lengths and were more prone to lodging than the other genotypes, but appeared to have higher grain yield. The *Hd16* genotype tended to have an increased culm length and a decreased protein content, but there was greater variability and number of panicles, while the 1,000-grain weight and grain yield tended to be lower. This is probably due to the late-maturing traits. This trend was probably influenced by the change of growing season to one more suited to the late trait. On the other hand, the introduction of *d60* by Koshihikari Hd16 resulted in a slight recovery of grain yield to the same levels as those of wild-type Koshihikari. It was verified that, even when cultivated for late maturity, the grain quality was maintained at the same level as that of wild-type Koshihikari, and the resistance to lodging trait was obtained by shortening the culm length.

Subsequently, analysis of cultivation areas suitable for the d60Hd16 line showed that Yamanashi and Ehime, which produced longer panicle and culm lengths, were better in terms of yield, while Shimane, which was warmer and produced shorter panicle and culm lengths, was better from the viewpoint of taste value and grain quality (**Figure 7B**).

### Multiple comparison analysis

**Figure 8:**
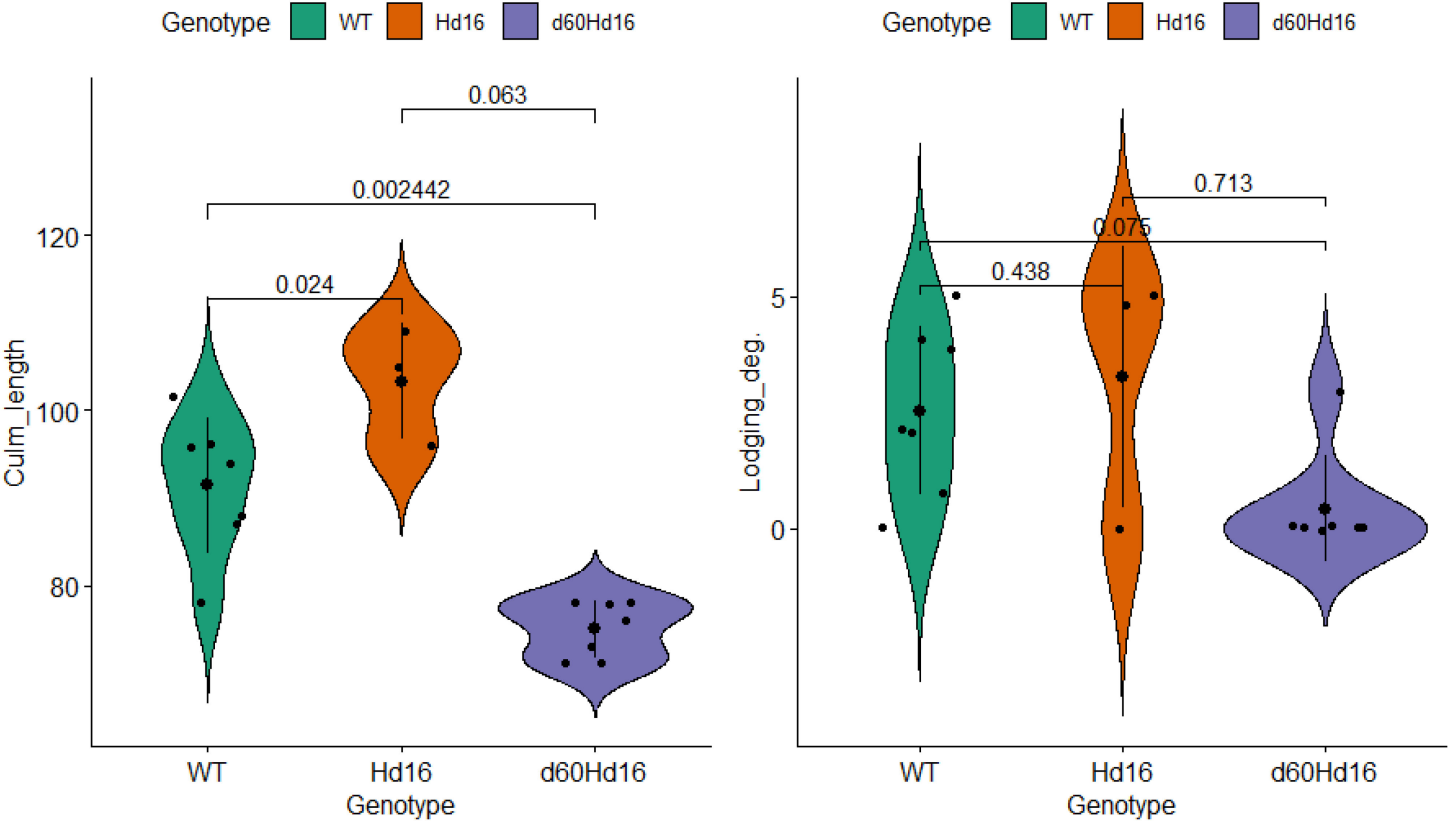
Comparison of traits among genotypes. *Small black points* indicate individual data. *Large points* and *bar* indicate the mean ± SD. Adjusted *p*-values using Holm’s method are shown on the *horizontal bar*. *Lodging_deg.*, degree of lodging. Degree of lodging was determined based on the inclination angle of the plant: 0, standing; 1, almost 70; 2, almost 50; 3, almost 30; 4, almost 10; and 5, lodged.

The distributions of each trait, environmental factor, and target trait were visualized in a violin plot, and statistically significant differences in the mean differences were analyzed (**Supplementary Figure S3**; **Figure 8**). To further confirm the short culm length and the low lodging degree of *d60Hd16* shown in the correlation and principal component analyses, the differences in the trait values between genotypes were examined. The results showed that culm length was significantly longer in *Hd16* (91.5 ± 7.7 cm) and significantly shorter in *d60Hd16* (75.0 ± 3.3 cm) compared with the wild type (91.5 ± 7.7 cm). This thereby indicated that a lodging degree was less likely to occur in *d60Hd16* compared with the wild type, although correction for multiplicity eliminated the significant difference (wild type: 2.5 ± 1.8; Hd16: 3.3 ± 2.8; d60Hd16: 0.43 ± 1.1). No other statistically significant differences in the target traits were found, suggesting that they are equivalent to the wild type. These results suggest that the Koshihikari d60Hd16 trait can be stably expressed in a wide range of regions in Japan.

## Discussion

The present study gathered four major conclusions, as follows:

1. Two rice genotypes, *Hd16* (late flowering) and *d60Hd16* (short culm and late flowering), were established in a homogeneous background of Koshihikari to improve resilience of Koshihikari against climate changes (**Figures 1**–**5**).
2. Quality was negatively correlated with panicle length and 1,000-grain weight, and yield was strongly positively correlated with 1,000-grain weight in the correlation and partial correlation analyses (**Figure 6**).
3. The *d60* genotype was negatively correlated with culm length and lodging degree and has the effect of improving the risk of collapse (increase of culm length) and low yield that might be caused by the *Hd16* genotype (**Figures 6**–**8**).
4. Suitable cultivation regions were suggested for each variety in the PCA (**Figure 7**).

The threat of strong typhoons, rainfall, and flood caused by global warming (Knutson et al., 2019) is causing serious lodging, consequent yield loss, and grain quality deterioration in rice production (Dahiya et al., 2018). Moreover, Koshihikari is suffering from poor filling and yield reduction caused by high-temperature maturation: preventing high-temperature damage in the hot summer is an effective solution to shift rice ripening to early autumn. Thus, in this study, the late-flowering gene *Hd16* from Koganebare was integrated into Koshihikari through four times of backcrossing (**Figure 2**). We consequently developed a semi-dwarf and late-flowering Koshihikari-type line (Koshihikari d60Hd16), the purposes of which were to stabilize high yield and to avoid high-temperature maturation (**Figures 3**, **4**), by crossing Koshihikari Hd16 with Koshihikari d60. WGS detected an SNP from adenine to guanine in the *Hd16* gene on chromosome 3, which was the same as a causative mutation of *Hd16* in Nipponbare (**Figure 5**). Furthermore, the majority of the genome was substituted to the Koshihikari genome, except for the *Hd16* region, in which there is a causative SNP of *Hd16*. These experimental processes demonstrated that a variety of developments occurred at the whole-genome level with high quality, and it is expected that good quality will be maintained in future cultivation. Furthermore, the SSR markers used in the experiments can be utilized in the quality testing of genotypes. As expected, Koshihikari d60Hd16 was 14.6 cm (16%) shorter and was 12.4 days later flowering than Koshihikari, indicating suitable traits for the threat of typhoons and high-temperature maturation. Remarkably, the yield of Koshihikari d60Hd16 (62.6 kg/a) was 6.0% higher than that of Koshihikari. Koshihikari d60Hd16 was registered as “Koshihikari Suruga d60Hd16” (Tomita, 2021).

Data from productivity tests have been used to understand the relationships between traits, qualities, cropping regions, and environmental factors (Ishizaki et al., 2005; Fukushima et al., 2015; Xu et al., 2015; Kobayashi et al., 2018; Li et al., 2019; Tiwari et al., 2019; TARI, 2020; Liang et al., 2021; Al-Daej, 2022; Ishimaru et al., 2022; Limbongan et al., 2023; Singh et al., 2023; Semeskandi et al., 2024). For instance, certain rice varieties show superior grain appearance under high-temperature conditions (Ishimaru et al., 2022). Al-Daej (2022) reported correlations between quality and the mineral element contents. Xu et al. (2015) showed that the 1,000-grain weight negatively affected quality, but was positively correlated with yield. In the present study, 1,000-grain weight was positively correlated with yield and was negatively correlated with quality; however, the partial correlation analysis suggested that its correlation with quality was indirect (no partial correlation was found) (**Figure 6**). Li et al. (2019) reported that the filled grain number per panicle, the plant height, panicle length, grains per panicle, long growth period, and the low panicle number accounted for the high yield. Plant height had a positive effect on the yield of the *indica* and *japonica* inbred, but had a negative effect on the *japonica* hybrid. In the present study, panicle length was not associated with the yield, and culm length and panicle number were positively related with yield only in the partial correlation (**Figure 6**). The correlations observed between multiple traits might only detect indirect correlations; however, to our knowledge, there are no reported relationships between traits examined using partial correlation. Thus, the present study stringently confirmed the correlation between the traits and environmental factors and provided fundamental knowledge for the planning of efficient cropping. The 1,000-grain weight is an important trait for estimating high yield, while the number of paddies and culm length are considered to be meaningful traits in terms of partial correlation.

In the present study, the breeding lines evaluated are suggested to express stable traits in a wide range of regions across the country (**Figure 7**). Although the sample size was very small, *Hd16* was related not only to late flowering but also to an increase of culm length. This observation was demonstrated by the partial correlation between this trait and culm length, suggesting that accumulated temperature could affect culm length (**Figure 6**). This is probably a secondary effect of the change in cropping season and that *Hd16* itself would not have the ability to control such traits. On the other hand, *d60* has the effect of improving the risk of collapse (increase of culm length) and low yield that could be caused by the *Hd16* genotype (**Figures 7**, **8**). The fact that the acquisition of *d60* maintained the late-flowering traits of *Hd16*, and improved the large inter-regional variations and low yields, is an interesting finding and suggests that the *d60* and *Hd16* combination is extremely meaningful for the development of new varieties to adapt to climate change.

The present study clearly showed the differences in the genotypes and cultivation regions using PCA (**Figure 7**). Similar analysis using PCA on the relationships among different strains of resistant near-isogenic lines of Koshihikari was conducted by Ishizaki et al. (2005), with these lines having similar agronomic traits, quality, and taste to the original variety. Moreover, Semeskandi et al. (2024) showed the benefits of combination analysis using PCA and correlation analysis for rice improvement cultivation. However, to our knowledge, only a few studies used PCA to identify differences among cultivation regions. Our results suggested suitable cultivation regions for each strain and found that Yamanashi and Ehime, which produce longer panicles, are suitable in terms of yield, while Shimane, which is warmer and produces shorter panicle and culm lengths, is preferable for taste value and grain quality. These results are understandable through the results of the correlation analysis in the present study (**Figure 6**). Taste value was negatively correlated with culm length and positively correlated with accumulated temperature using partial correlation. Grain quality was positively correlated with accumulated temperature and negatively correlated with panicle length and 1,000-grain weight. Thus, in warmer regions, the taste value and quality are higher, while yield is lower.

Partial correlation analysis and network analysis are useful for discussing the relationships between factors; however, they do not directly reveal causal relationships. Therefore, further research is needed to clarify the mechanistic relationships between genotypes and traits. Moreover, the present study does not clarify the differences in traits in regions with similar accumulated temperatures. The relationship between environmental factors and traits has been investigated, and some studies have highlighted the importance of environmental conditions in determining yield and quality. Genotypic and phenotypic correlations have been noted (Tiwari et al., 2019), as well as the impact of high temperatures on grain appearance (Ishimaru et al., 2022) and the effect of water consumption and rainfall on yield (Semeskandi et al., 2024). Therefore, the inclusion of various environmental factors, such as precipitation, to the new analysis items could provide a more in-depth understanding of regional factors influencing traits. In addition, as the yield in this study was a calculated value, accurate measured data will improve the analytical accuracy. If a link between genotype and trait, as well as various environmental factors, is found, it is expected that quantitative simulation of yield in new growing areas, e.g., by modeling the relationship, can be carried out. For the prediction of traits, it is important to expand the spatiotemporal data, and it is necessary to obtain data on the different climatic conditions at the same location, as well as regional characteristics. Therefore, continuous analysis in randomized blocks with multi-year cropping would be important.

## Conclusions

In conclusion, productivity tests of the Koshihikari homologous genotypes, the late-flowering *Hd16* and the short-statured, late-flowering *d60Hd16*, were conducted in nine prefectures in Japan. The relationships between genotypes, traits, and temperature accumulation were analyzed. The *d60* and *Hd16* genotypes expressed stable traits adapted to a wide range of Japanese climatic conditions and growing environments. Furthermore, the results of various biostatistical analyses showed that quality was negatively correlated with panicle length and 1,000-grain weight and that yield was strongly positively correlated with 1,000-grain weight. The *d60* genotype was negatively correlated with culm length and lodging degree. *Hd16* adds late-flowering traits to rice and has a limited effect on other visible traits. However, partial correlation could potentially have the effect of increasing the culm length and making it more prone to collapse. This negative aspect was found to be completely suppressed by the effect of *d60* shortening the culm length, making it less prone to collapse in the d60Hd16 line. In addition, *d60* causes negative correlations with taste value and the protein content; however, these are considered to be secondary effects via the lower culm length.

The results of the PCA showed that, in Koshihikari d60Hd16, Yamanashi and Ehime, which produce longer panicle and culm lengths, are better in terms of yield, while Shimane, which is warmer and produces shorter ear and culm lengths, is better in terms of taste value and grain quality. The results also suggest that Koshihikari d60Hd16 can express traits that make it less susceptible to lodging degree while maintaining the same quality and yield as that of the wild type when grown as a late-flowering line. This study will provide fundamental information for the promotion of smart agriculture using improved varieties.

## Data Availability

The original contributions presented in the study are publicly available. This data can be found here: https://figshare.com/articles/dataset/rice_d60_variants_vcf_gz_vcf/28431098?file=52420808.

## Supplementary material

Supplementary Figure 1 Prefectures in this study. Productivity tests were conducted at Research Institute of Environment, Agriculture and Fisheries, Osaka Prefecture (Habikino city, Osaka), Mie Prefectural Agricultural Research Institute (Matsusaka city, Mie), Kochi Agricultural Research Center (Nankoku city, Kochi), Shimane Agricultural Technology Center (Izumo city, Shimane), Ehime Research Institute of Agriculture, Forestry and Fisheries (Matsuyama city, Ehime), Saga Prefectural Agriculture Research Center (Saga city, Saga), and Miyagi Prefectural Furukawa Agricultural Experiment Station (Osaki city, Miyagi).

Supplementary Figure 2 Scatter plots and correlation analysis. WT: wildtype, Panicles_no.: No. of panicles, Lodging_deg.: Lodging degree, 1000_grain_wt: 1,000-grain weight, Protein_cont.: Protein content, Accum.temp.: Accumulated temperature. **(A)** all strains, **(B)** *Hd16*, **(C)** *d60Hd16.*

Supplementary Figure 3 Comparison of traits, environmental factors, and target traits among genotype. WT: wildtype, Panicles_no.: No. of panicles, Lodging_deg.: Lodging degree, 1000_grain_wt: 1,000_grain_weight, Protein_cont.: Protein content, Accum.temp.: Accumulated temperature.

Supplementary Table 1 Summary of productivity test. WT: wildtype, Panicles_no.: No. of panicles, Lodging_deg.: Lodging degree, 1000_grain_wt: 1,000-grain weight (g), Protein_cont.: Protein content (%), Accum.temp.: Accumulated temperature (°C). Productivity tests were conducted in Miyagi, Yamanashi, Shizuoka, Mie, Osaka, Shimane, Ehime, Kochi and Saga prefectures for Koshihikari, Koshihikari Hd16 and Koshihikari d60Hd16 to evaluate the following parameters: culm length (cm), panicle length (cm), number of panicles (/m^2^), lodging degree, 1,000 grain weight (g), protein content (%), accumulated temperature (°C), grain yield (kg/a), grain quality and value of taste. Lodging degree was determined based on the inclination angle of plant; 0: standing, 1: almost 70, 2: almost 50, 3: almost 30, 4: almost 10, 5: lodged. Grain quality was classified into nine grades; 1: excellent good to 9: especially bad low quality. Value of taste were evaluated as seven grades of organoleptic assessment by panelist.
