# Supplementary figures and images for: A semi-dwarf and late-flowering Koshihikari d60Hd16: development, productivity, and regional suitability revealed by correlation-based network analysis
Source: Front Plant Sci. 2025 Mar 3;15:1443149. doi: 10.3389/fpls.2024.1443149 (PMC11912565; doi:10.3389/fpls.2024.1443149)

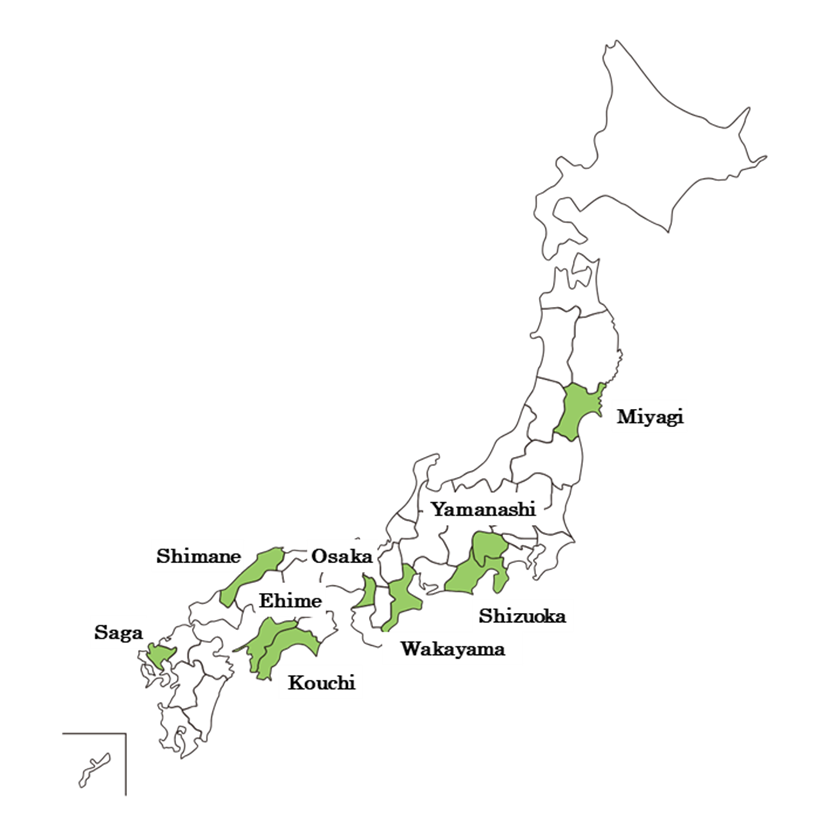

Supplement: Supplementary Figure 1 — Prefectures in this study. Productivity tests were conducted at Research Institute of Environment, Agriculture and Fisheries, Osaka Prefecture (Habikino city, Osaka), Mie Prefectural Agricultural Research Institute (Matsusaka city, Mie), Kochi Agricultural Research Center (Nankoku city, Kochi), Shimane Agricultural Technology Center (Izumo city, Shimane), Ehime Research Institute of Agriculture, Forestry and Fisheries (Matsuyama city, Ehime), Saga Prefectural Agriculture Research Center (Saga city, Saga), and Miyagi Prefectural Furukawa Agricultural Experiment Station (Osaki city, Miyagi). [file Image1.tif]

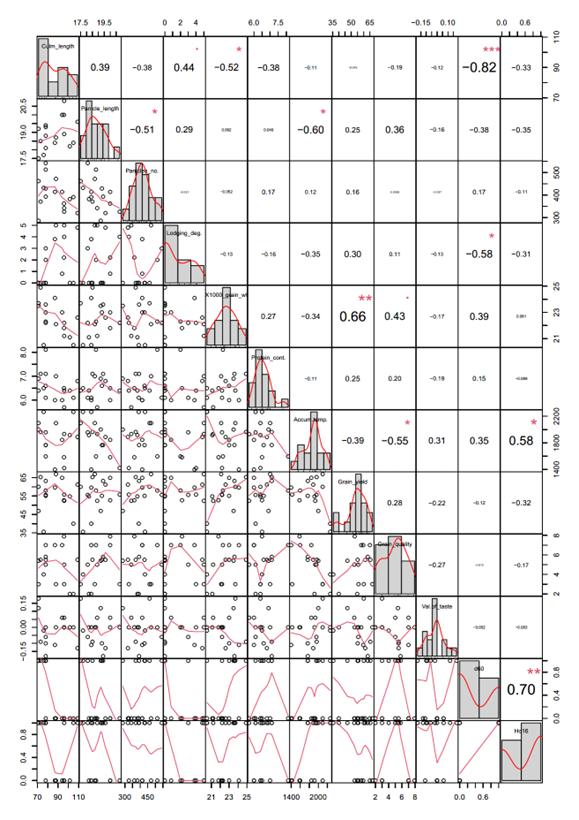

Supplement: Supplementary Figure 2 — Scatter plots and correlation analysis. WT: wildtype, Panicles_no.: No. of panicles, Lodging_deg.: Lodging degree, 1000_grain_wt: 1,000-grain weight, Protein_cont.: Protein content, Accum.temp.: Accumulated temperature. (A) all strains, (B) Hd16, (C) d60Hd16. [file Image2.tif]

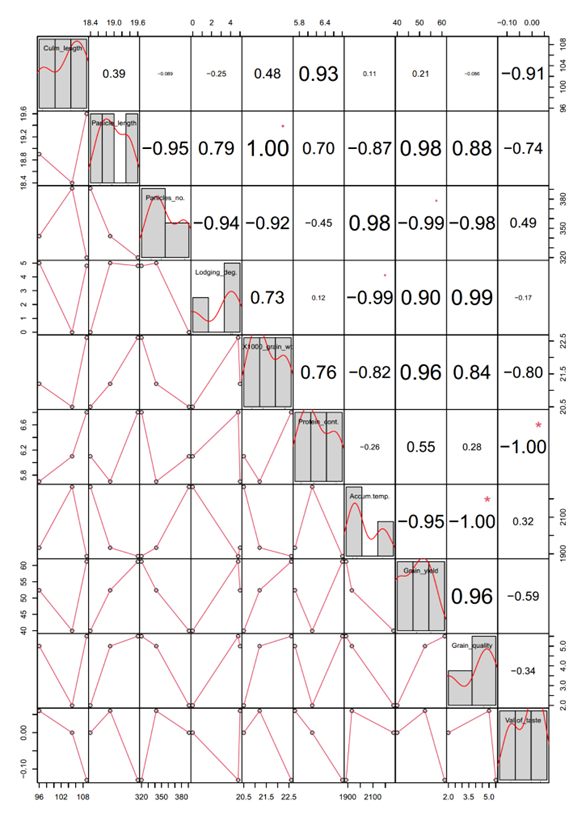

Supplement: Supplementary Figure 3 — Comparison of traits, environmental factors, and target traits among genotype. WT: wildtype, Panicles_no.: No. of panicles, Lodging_deg.: Lodging degree, 1000_grain_wt: 1,000_grain_weight, Protein_cont.: Protein content, Accum.temp.: Accumulated temperature. [file Image3.tif]

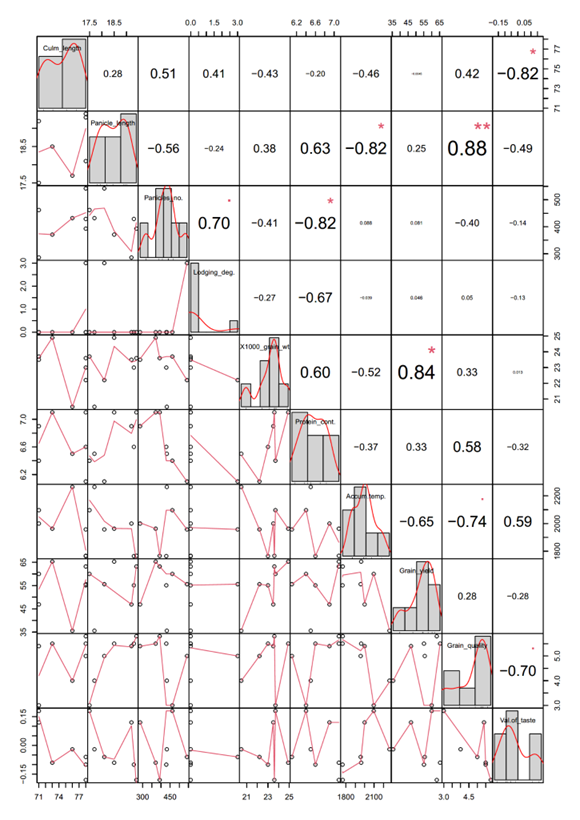

Supplement: Supplementary file 4 [file Image4.tif]

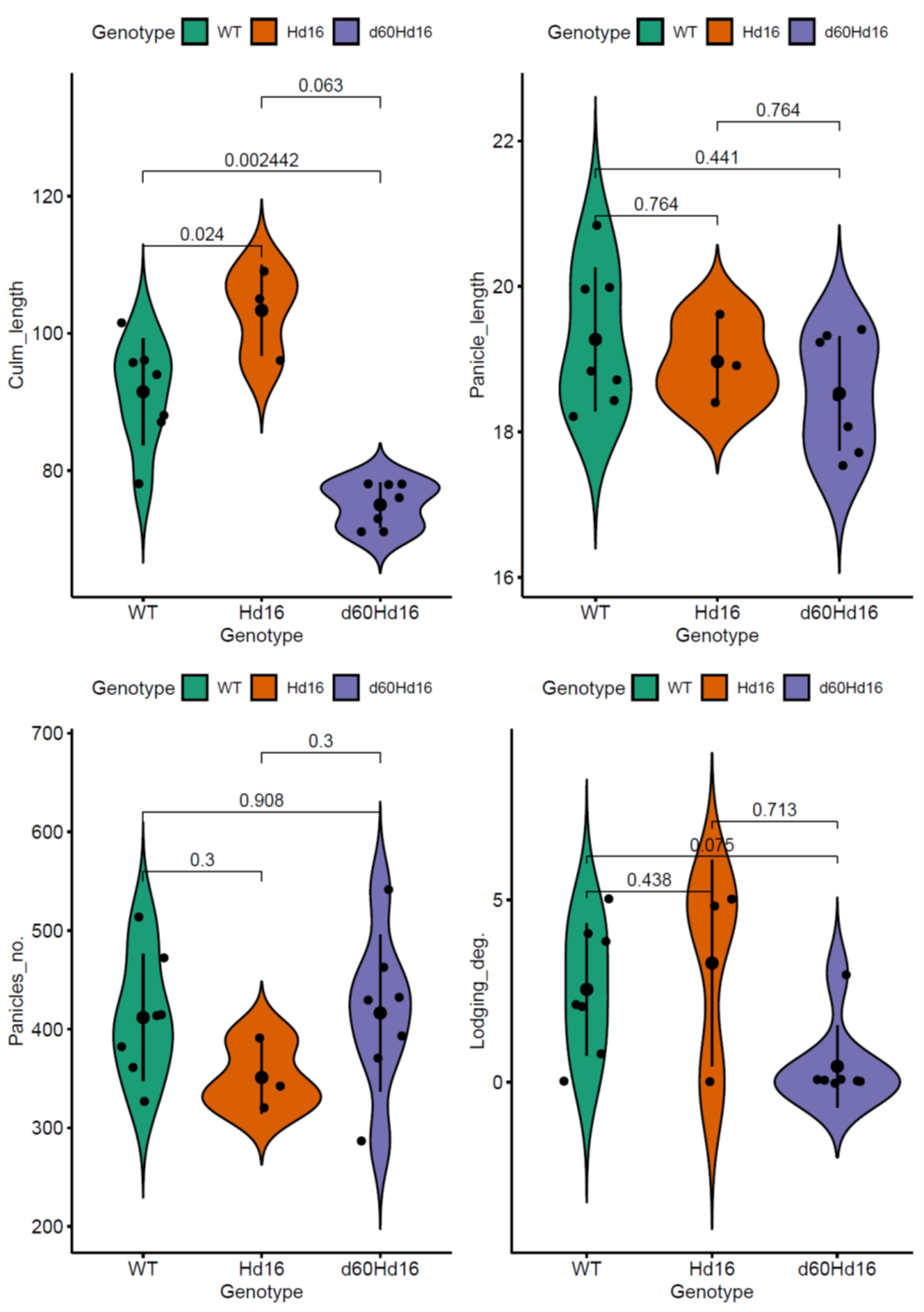

Supplement: Supplementary file 5 [file Image5.tif]

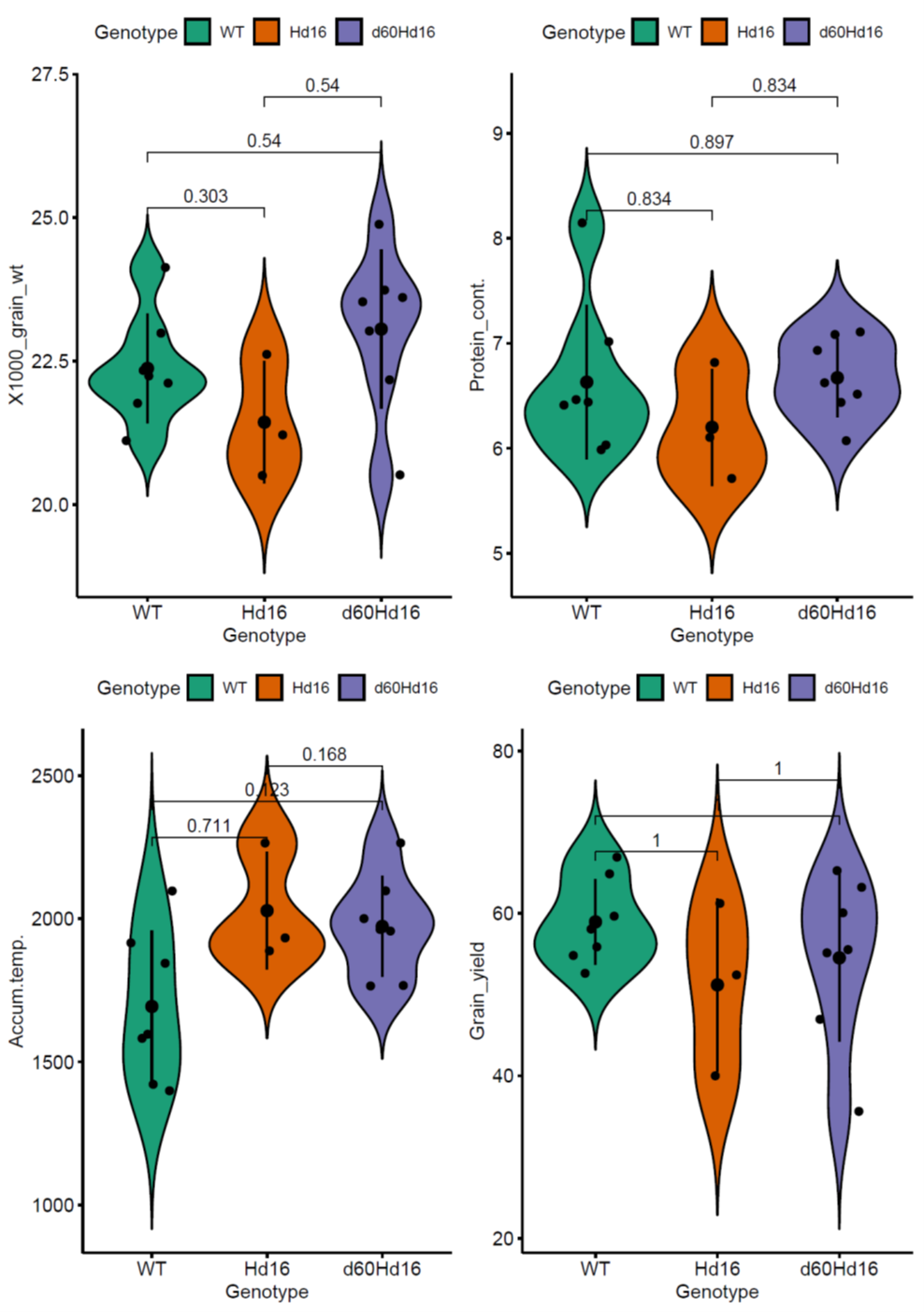

Supplement: Supplementary file 6 [file Image6.tif]

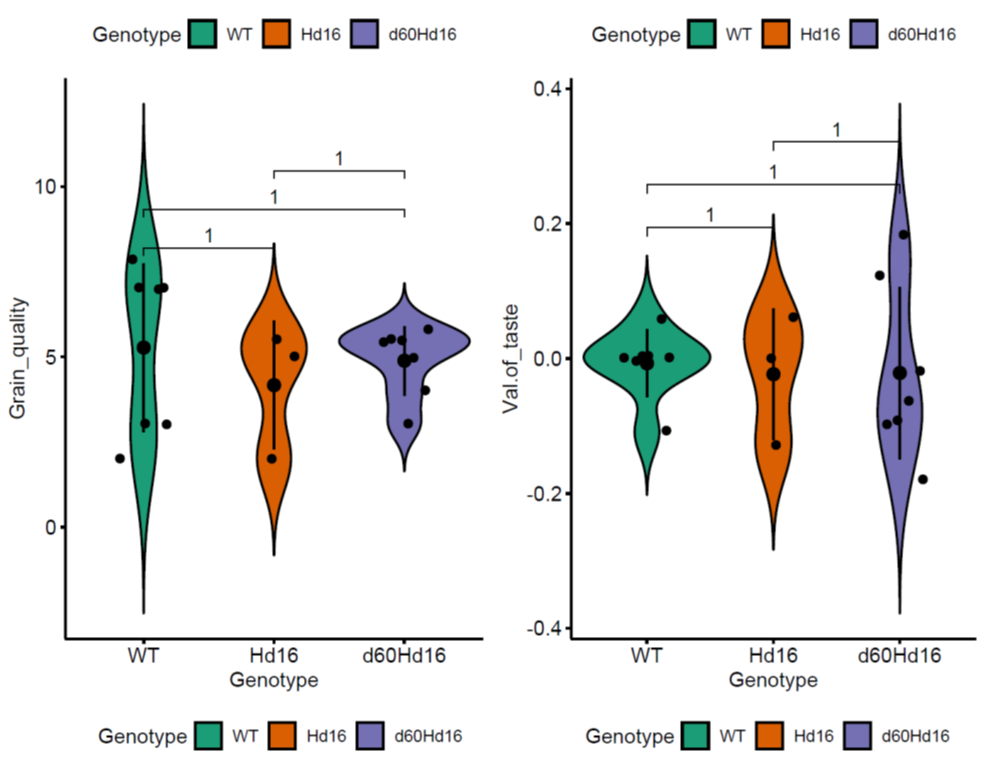

Supplement: Supplementary file 7 [file Image7.tif]
