# Supplementary material for: A semi-dwarf and late-flowering Koshihikari d60Hd16: development, productivity, and regional suitability revealed by correlation-based network analysis
Source: Front Plant Sci. 2025 Mar 3;15:1443149. doi: 10.3389/fpls.2024.1443149 (PMC11912565; doi:10.3389/fpls.2024.1443149)
Supplement: Supplementary Table 1 — Summary of productivity test. WT: wildtype, Panicles_no.: No. of panicles, Lodging_deg.: Lodging degree, 1000_grain_wt: 1,000-grain weight (g), Protein_cont.: Protein content (%), Accum.temp.: Accumulated temperature (°C). Productivity tests were conducted in Miyagi, Yamanashi, Shizuoka, Mie, Osaka, Shimane, Ehime, Kochi and Saga prefectures for Koshihikari, Koshihikari Hd16 and Koshihikari d60Hd16 to evaluate the following parameters: culm length (cm), panicle length (cm), number of panicles (/m2), lodging degree, 1,000 grain weight (g), protein content (%), accumulated temperature (°C), grain yield (kg/a), grain quality and value of taste. Lodging degree was determined based on the inclination angle of plant; 0: standing, 1: almost 70, 2: almost 50, 3: almost 30, 4: almost 10, 5: lodged. Grain quality was classified into nine grades; 1: excellent good to 9: especially bad low quality. Value of taste were evaluated as seven grades of organoleptic assessment by panelist. [file Table1.docx]

**Supplementary Material**

Supplementary table S1. Summary of productivity test.


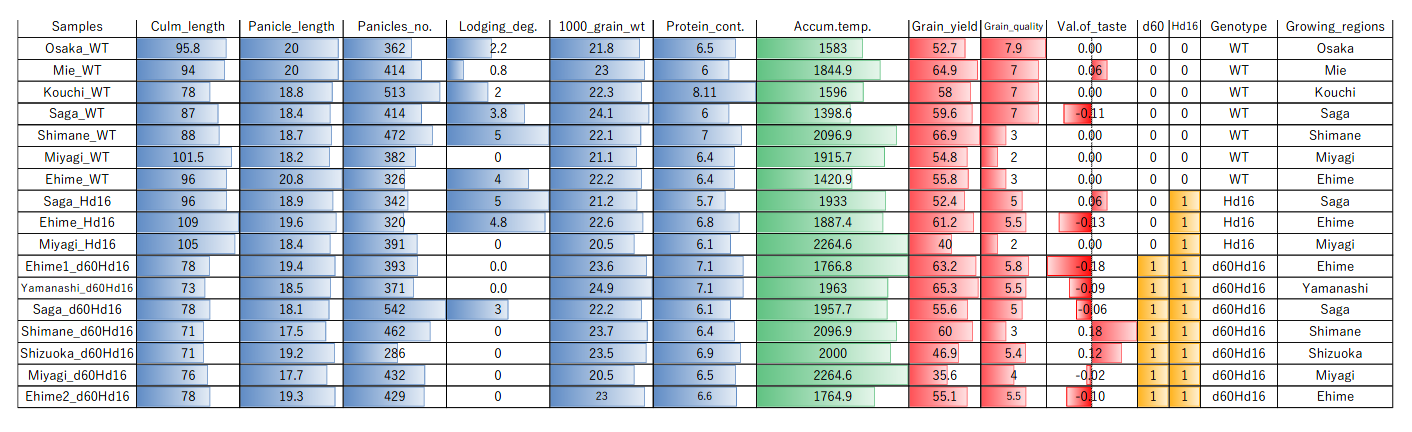


WT: wildtype, Panicles_no.: No. of panicles, Lodging_deg.: Lodging degree, 1000_grain_wt: 1,000-grain weight (g), Protein_cont.: Protein content (%), Accum.temp.: Accumulated temperature (℃)

Productivity tests were conducted in Miyagi, Yamanashi, Shizuoka, Mie, Osaka, Shimane, Ehime, Kochi and Saga prefectures for Koshihikari, Koshihikari Hd16 and Koshihikari d60Hd16 to evaluate the following parameters: culm length (㎝), panicle length (㎝), number of panicles (/㎡), lodging degree, 1,000 grain weight (g), protein content (%), accumulated temperature (℃), grain yield (kg/a), grain quality and value of taste. Lodging degree was determined based on the inclination angle of plant; 0: standing, 1: almost 70, 2: almost 50, 3: almost 30, 4: almost 10, 5: lodged. Grain quality was classified into nine grades; 1: excellent good to 9: especially bad low quality. Value of taste were evaluated as seven grades of organoleptic assessment by panelist.
